# Algorithm-Enabled, Personalized Glucose Management for Type 1 Diabetes at the Population Scale: Prospective Evaluation in Clinical Practice

**DOI:** 10.2196/27284

**Published:** 2022-06-06

**Authors:** David Scheinker, Angela Gu, Joshua Grossman, Andrew Ward, Oseas Ayerdi, Daniel Miller, Jeannine Leverenz, Korey Hood, Ming Yeh Lee, David M Maahs, Priya Prahalad

**Affiliations:** 1 Department of Pediatrics, Division of Pediatric Endocrinology Stanford University Stanford, CA United States; 2 Lucile Packard Children's Hospital Stanford University Stanford, CA United States; 3 Department of Management Science and Engineering Stanford University School of Engineering Stanford, CA United States; 4 Stanford Diabetes Research Center Stanford University Stanford, CA United States; 5 Department of Health Research and Policy Stanford University Stanford, CA United States

**Keywords:** telehealth, diabetes, population health, continuous glucose monitor, personalized medicine

## Abstract

**Background:**

The use of continuous glucose monitors (CGMs) is recommended as the standard of care by the American Diabetes Association for individuals with type 1 diabetes (T1D). Few hardware-agnostic, open-source, whole-population tools are available to facilitate the use of CGM data by clinicians such as physicians and certified diabetes educators.

**Objective:**

This study aimed to develop a tool that identifies patients appropriate for contact using an asynchronous message through electronic medical records while minimizing the number of patients reviewed by a certified diabetes educator or physician using the tool.

**Methods:**

We used consensus guidelines to develop timely interventions for diabetes excellence (TIDE), an open-source hardware-agnostic tool to analyze CGM data to identify patients with deteriorating glucose control by generating generic flags (eg, mean glucose [MG] >170 mg/dL) and personalized flags (eg, MG increased by >10 mg/dL). In a prospective 7-week study in a pediatric T1D clinic, we measured the sensitivity of TIDE in identifying patients appropriate for contact and the number of patients reviewed. We simulated measures of the workload generated by TIDE, including the average number of time in range (TIR) flags per patient per review period, on a convenience sample of eight external data sets, 6 from clinical trials and 2 donated by research foundations.

**Results:**

Over the 7 weeks of evaluation, the clinical population increased from 56 to 64 patients. The mean sensitivity was 99% (242/245; SD 2.5%), and the mean reduction in the number of patients reviewed was 42.6% (182/427; SD 10.9%). The 8 external data sets contained 1365 patients with 30,017 weeks of data collected by 7 types of CGMs. The rates of generic and personalized TIR flags per patient per review period were, respectively, 0.15 and 0.12 in the data set with the lowest average MG (141 mg/dL) and 0.95 and 0.22 in the data set with the highest average MG (207 mg/dL).

**Conclusions:**

TIDE is an open-source hardware-agnostic tool for personalized analysis of CGM data at the clinical population scale. In a pediatric T1D clinic, TIDE identified 99% of patients appropriate for contact using an asynchronous message through electronic medical records while reducing the number of patients reviewed by certified diabetes care and education specialists by 43%. For each of the 8 external data sets, simulation of the use of TIDE produced fewer than 0.25 personalized TIR flags per patient per review period. The use of TIDE to support telemedicine-based T1D care may facilitate sensitive and efficient guideline-based population health management.

## Introduction

### Background

For patients with type 1 diabetes (T1D) receiving insulin therapy, the American Diabetes Association (ADA) recommends the use of continuous glucose monitors (CGMs) as the standard of care along with quarterly clinic visits with hemoglobin A_1c_ (HbA_1c_) laboratory testing [1]. However, most people with T1D remain on self-monitored blood glucose because of patient, clinician, or insurance preference and do not meet the current HbA_1c_ targets [2]. The long feedback cycle and the use of relatively little data when self-monitoring are barriers to timely detection and personalized response to deteriorating glucose control. An individual self-monitoring their glucose levels in line with the 2018 ADA standard of care recommendations generates glucose readings 6-10 times per day and receives feedback from their care team every 1-4 months based on a clinic visit or an HbA_1c_ test [3]. In contrast, CGMs record glucose levels once every 5-15 minutes (96-288 times per day). The initiation and continued use of CGMs have increased and are associated with improved clinical outcomes and patient-reported quality of life measures [1-9]. In a US pediatric T1D registry, the use of CGMs increased from 4% in 2013 to 33% in 2017 [6].

Numerous commercial and open-source platforms provide individual-level visualizations and analyses of CGM data [10-13]. Recent studies by the Advanced Technologies and Treatments for Diabetes consensus on CGM and the ADA and European Association for the Study of Diabetes consensus on precision medicine in diabetes found that although the use of CGMs offers an opportunity to use high-frequency data to identify deteriorating glucose control and tailor personalized management strategies, no standardized, validated methods currently exist outside of automated insulin delivery systems [4,14]. Patient-level tools, such as manufacturer or data aggregator platforms, require physicians or certified diabetes care and education specialists (CDCESs) to examine the data of each individual to identify those people whose glucose management may need improvement. Population-level tools that analyze and present data for the entire population to facilitate prioritizing patients are less common. LibreView (Abbott Laboratories) enables whole-population data review but is proprietary, works only with Libre sensors, and provides access primarily to prespecified metrics [12]. A hardware-agnostic tool would be more appropriate to support care for a population in which patients use a variety of CGMs. An open-source tool would facilitate external evaluation and the development and comparison of alternative models. A tool that calculates *personalized* metrics for each patient based on their historical data would facilitate the tracking of temporal changes in glucose management. To the best of our knowledge, no validated, hardware-agnostic, open-source tool is available to facilitate the delivery of timely, population-level, personalized care through telehealth.

Numerous efforts have been made to improve T1D management using remote monitoring, the most successful of which relied on asynchronous messages sent to patients [15]. However, not all studies demonstrated significant and sustained improvement [15]. The implementation of clinical decision support (CDS) has faced a variety of challenges and has led to structured recommendations for their successful design and deployment [16]. Two of the primary areas of focus are that the CDS should improve, rather than disrupt, the appropriate workflows and that it should be designed with an iterative approach [16]. A recent multi-institution, cluster randomized clinical trial on the use of a CDS to improve the management of heart disease showed no significant improvements [17]. There was insufficient evaluation and redesign of the system based on feedback from its intended users [18]. The lack of iterative design and the resulting challenges to the workflow are common in the design of clinical software following the waterfall approach, a structured top-down approach in which the intent is to test and finalize the tool before deployment [19]. The agile approach, a more iterative approach based on rapid deployment and iterative redesign, is a popular alternative [19].

### Objectives

We sought to design timely interventions for diabetes excellence (TIDE), a decision support tool to identify patients appropriate for asynchronous contact using a secure message through electronic medical record (EMR). To facilitate successful deployment and sustained use, we sought to fit into and improve current workflows by reducing the number of patients requiring review by a physician or the CDCES. We followed the agile approach to deploy the earliest viable version of TIDE in clinical practice and update it based on feedback from physicians and CDCESs.

## Methods

### Study Design

This study followed the Guidelines for Developing and Reporting Machine Learning Predictive Models in Biomedical Research [20]. The first phase was hardware-agnostic algorithm design based on the data collected using a variety of CGM hardware. Data were obtained from a convenience sample of eight external data sets: 6 from clinical trials and 2 donated by research foundations [21-28]. The second phase was the design of TIDE, an interactive visual interface presenting CGM data for the entire clinical population, based on iterative feedback from physicians and CDCESs at an academic pediatric T1D clinic. One physician and CDCES used TIDE for 4 weeks and provided feedback based on which TIDE was comprehensively redesigned. Following this, they used the redesigned version for 5 weeks, during which minor improvements were made and errors were fixed. The third phase was a prospective evaluation over the course of 7 weeks of the sensitivity of TIDE for identifying patients who are appropriate for asynchronous contact using a secure message through the EMR and the difference in the number of patients requiring review with and without the use of TIDE. In the final phase, we simulated several measures of the workload generated by TIDE on the same convenience sample of 8 external data sets used to design the algorithms.

### Setting

TIDE was developed at an academic pediatric T1D clinic caring for youth newly diagnosed with T1D who initiated CGM use within 1 month of onset and enrolled in a weekly remote monitoring program. All patients used Dexcom G6 (Dexcom) monitors, from which data were uploaded and made available to physicians and CDCESs through the Dexcom Clarity Clinic Portal [10]. Participants consented to participate in a longitudinal study evaluating the initiation of CGM early in the course of diabetes and the effects of weekly CGM data review as part of a larger ongoing study, for which the details of the consent process, eligibility criteria, screening, and enrollment process have been reported [8]. Each week, a CDCES used the Dexcom Clarity Clinic Portal to review each patient’s data and send an asynchronous message through the EMRs to those patients who they determined required glucose management guidance. This study was approved by the Stanford University institutional review board. The leadership of the clinic, study authors PP and DMM, who serve on relevant national organizations governing diabetes technology, approved the use of TIDE in clinical care.

### Generic and Personalized Metrics

A metric is *generic* if it is calculated the same way for each patient, for example, mean glucose (MG), and *personalized* if it is calculated for each patient based on their historical data; for example, the month-to-month change in MG. Consensus guidelines were used to generate a large set of generic CGM-based metrics from which clinicians could select the metrics to be tracked in TIDE [29]. The metrics included the number of days the CGM was active and collected more than a minimum percentage of valid readings (ACT), MG, percentage of time in range (TIR) defined as readings 70 to 180 mg/dL, percentage of time extremely hypoglycemic (eHyp) defined as readings <54 mg/dL, and percentage of time hypoglycemic (Hyp) defined as readings <70 mg/dL. Following the same consensus guidelines, each metric was calculated for each day for the entire day from 0:00 to 24:00, daytime 6:00 AM to midnight, and nighttime midnight to 6:00 AM. A complete list of generic metrics is provided in Table S1 in Multimedia Appendix 1. For each generic metric, a personalized metric was defined as the change from a baseline period to a review period (eg, the month-to-month change in the MG). The *review period* is the timeframe over which the metrics are calculated. It is defined relative to the day on which the data are being reviewed (eg, the last full week). The *baseline period* is the timeframe over which the baseline value for each personalized metric is calculated (eg, the last full month before the review period). For each metric, “a flag is triggered” when the metric exceeds a prespecified target value.

Algorithms to calculate generic and personalized metrics and to generate flags as a function of the review period, baseline period, and target value were developed and tested using data from 8 external data sets. The data sets were identified based on an internet search and the professional contacts of the authors: 6 previously published clinical trials and observational data donated by Tidepool and OpenAPS (Table S2 in Multimedia Appendix 1). Data use agreements were signed with Tidepool and OpenAPS, each stipulating that the data may be used for this research project and that those donating the data would not take part in the study design or the reporting of results and would be acknowledged in writing. The data sets had 168,723 patient days of included CGM readings collected with seven types of CGMs: Freestyle Navigator, Dexcom STS, Medtronic Paradigm or Guardian, iPro2, iPro2 Professional CGM, Freestyle Libre Pro Flash, and Dexcom G4. The algorithms with annotated codes and an overview of their design, TIDE, and synthetic CGM data for use with TIDE are available on GitHub [30,31].

### Iterative Design of an Interactive Tool

The design of an initial version of TIDE was based on a convenience sample of informal interviews and observations used to establish the current state, achieve buy-in from stakeholders, and solicit suggestions for and perceived problems with the proposed workflow. The initial version of TIDE was designed to require a one-time setup followed by repeated use. During the one-time setup, based on their clinical practice and population, clinicians select the metrics to be displayed, the review period over which the generic metrics are calculated, and a baseline period based on which the personalized metrics are calculated. Two pediatric endocrinologists, study authors DMM and PP, and a CDCES, study author JL, identified the consensus glucose metrics currently being used in the clinic to evaluate patient glucose management: ACT was measured as the number of valid readings as a percentage of the maximum number of readings possible (the number of 5-minute intervals during the review period), MG, TIR, eHyp, and Hyp. The review period was set to 1 week ending on the last Sunday before the data review. The targets were initially set as follows: ACT >75%, TIR >70%, eHyp <1%, and Hyp <4%. No target was set for the MG. The valid wear threshold was used by the CDCES to determine whether to reach out to the patient to discuss their use of the CGM and to assist with challenges in obtaining additional sensors. When the valid wear threshold was not met, TIDE presented the metrics and flags as usual, and the CDCES used their judgment to determine whether patient data required further review.

During each of the first 4 weeks of the use of TIDE, the physician and study author PP logged into the Dexcom Clarity Clinic Portal and downloaded patient ID numbers, CGM readings, and CGM timestamps for all patients in the study. The physician used TIDE to identify patients with flags, reviewed the output of TIDE for appropriateness and patient safety, and forwarded the list of patients flagged by TIDE to CDCES (study author JL). For each flagged patient, the CDCES opened Dexcom Clarity and reviewed detailed patient data, opened the EMR and sent a secure message to the patient, and, if a dose adjustment was made, the dose was updated in the patient’s chart. After these 4 weeks, PP and JL suggested changes that were incorporated into TIDE between weeks 4 and 5. During weeks 5 to 9, the tool was used by the study authors PP and JL to provide clinical care and identify minor adjustments required to improve usability or correct errors. Minor adjustments and corrections were made immediately, usually within 24 hours of identification.

### Prospective Evaluation

The primary measures were the sensitivity of TIDE for identifying patients appropriate for asynchronous contact using a secure message through EMRs and the reduction in the number of patients reviewed by the CDCES with the use of TIDE. For 7 weeks, a CDCES reviewed the CGM data of each patient in the population and determined those appropriate for asynchronous contact. Sensitivity was defined as the number of appropriate patients flagged by TIDE divided by the number of appropriate patients. The reduction in the number of patients reviewed using TIDE was the number of patients not flagged by TIDE as a percentage of the total population.

The secondary outcomes calculated were as follows: the average amount of time required for per-patient review and contact as measured by the CDCES using TIDE, specificity (the number of patients not flagged by TIDE divided by the number of patients not appropriate for asynchronous contact), positive predictive power (the number of patients appropriately flagged by TIDE divided the number of patients flagged by TIDE), and negative predictive power (the number of patients not appropriate for asynchronous contact not flagged by TIDE divided by the number of patients not flagged by TIDE).

### Validation on External Data Sets

The primary determinant of the workload associated with the use of TIDE is the number of patients for whom flags are generated in each review period, equivalent to the rate at which flags are generated per patient per review period. To evaluate the workload associated with the use of TIDE in other settings, we simulated the rate at which generic and personalized flags would be generated for populations with varying levels of glucose management. Patient ID numbers, CGM readings, and CGM timestamps were extracted from each of the 8 external data sets for all patient days that met the inclusion criteria and at least 70% valid CGM readings. Metrics MG, TIR, eHyp, and Hyp Flag and the thresholds for these metrics were chosen based on consensus guidelines [29]. The thresholds for generic metrics were MG >170 mg/dL, TIR <60%, eHyp >1%, and Hyp >3%. Flag thresholds for personalized metrics were chosen based on the clinical experience of the study authors DMM and PP, as TIR less than baseline TIR minus 10 percentage points and MG greater than baseline MG plus 10 mg/dL. The duration of each measurement period was 1 week starting on Sunday. Personalized flags were calculated for patients with at least 4 weeks of data. For each personalized metric, the baseline period was the last week in the patient data that preceded the week being analyzed. The rate at which flags were generated was measured for each type of flag, each patient, and each dataset. For each data set, the rates at which flags were generated and the percentage of patients for whom at least one MG or TIR flag was generated every week were calculated. The rates of generic and personalized flags for MG and TIR were compared using a 2-tailed paired *t* test. The number of patients for whom an MG or TIR flag was generated every week was recorded. 

## Results

### Iterative Design of an Interactive Tool

**Figure 1:**
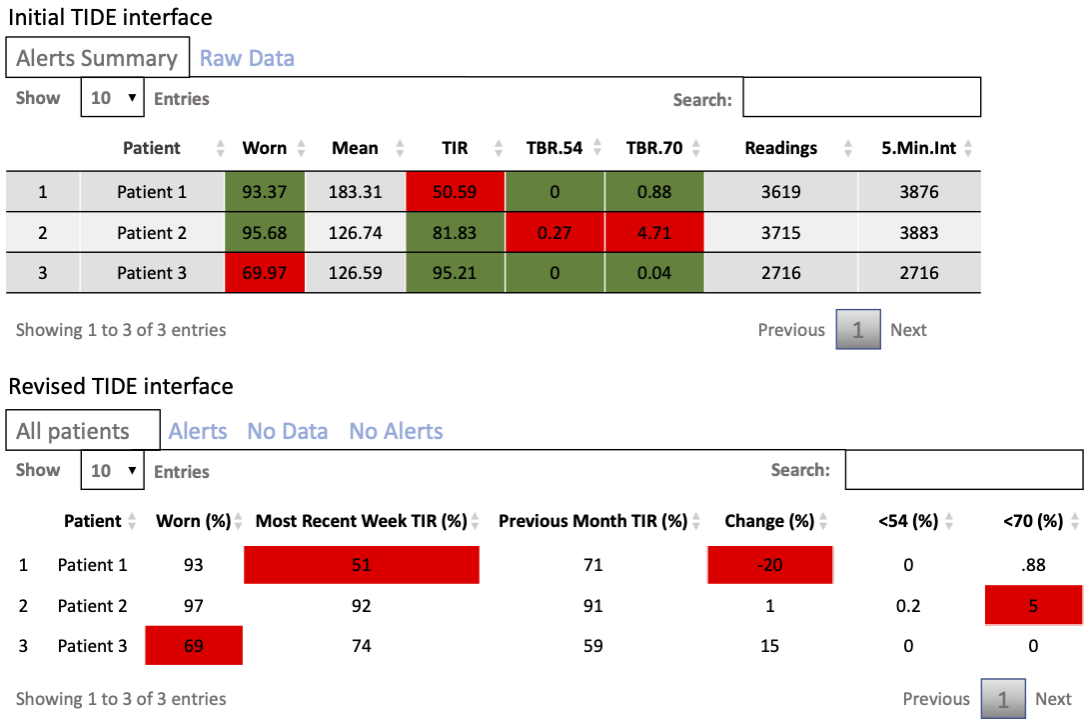
Initial and revised timely interventions for diabetes excellence (TIDE) interface. TBR: time below range; TIR: time in range.

The first version of TIDE displayed the metrics that triggered a flag in red and those that did not trigger a flag in green (Figure 1). After the feedback from the first 4 weeks of its use, the primary changes to the interface were as follows: the columns displaying MG, the number of readings, and the number of 5-minute intervals during the review period were removed to minimize the number of patients who received flags that did not require dose adjustments while not missing those who required dose adjustments; the criterion for TIR target was changed from 70% to 60%; a personalized metric was added to compare each patient’s TIR in the previous week to their TIR in the previous 4 weeks with a target of an increase in TIR or a drop in TIR of no more than 10% points; the color-coding was revised so only metrics that triggered a flag were highlighted; the wording of the display names of the metrics was changed to be more interpretable; a feature was added to allow the person using the tool to specify whether to use data from the most recent 7 days or from the default review period of 7 days ending on the previous Sunday; the patient data, previously presented on a single tab, were split tab into four tabs that displayed all patients, patients with alerts, patients with no data, and patients with data but no alerts; and the visual presentation was made more compact to display more patients per page (Figure 1).

**Figure 2:**
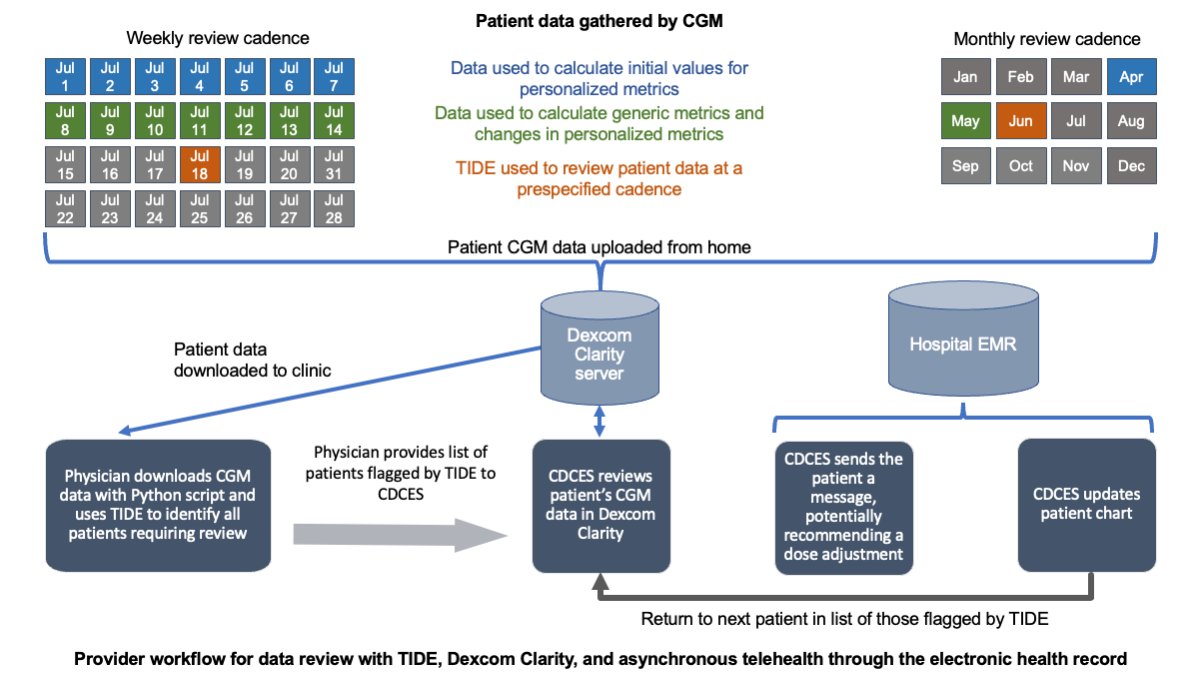
Workflow illustrated with examples of weekly and monthly cadence of data collection, data transfer, and provider review. CDCES: certified diabetes care and education specialists; CGM: continuous glucose monitor; EMR: electronic medical record; TIDE: timely interventions for diabetes excellence.

The primary change in the workflow was that the step of downloading each patient’s data from Dexcom Clarity was replaced with a Python script that downloaded all patient data (Figure 2). The participation of the physician in the review process was no longer necessary as part of the workflow but was continued to ensure patient safety and quality of care. The ultimate intended workflow, initiated months after the completion of this study, is for the CDCES to use TIDE without the participation of physicians.

### Prospective Evaluation

Over the last 7 weeks of the study, the number of patients increased from 56 to 64, totaling 427 patient weeks. The sensitivity of TIDE for identifying patients appropriate for contact using an asynchronous message through the EMR was 94% in the first week, 96% in the last week, and 100% in all other weeks (mean 99%, SD 2.5%; Table 1). The average reduction in the number of patients reviewed by the CDCES was 42.8% (182/427; SD 10.9%), that is, the fraction of patients not flagged for review by TIDE (Table 1).

For patients identified by TIDE as requiring review, the mean duration of the data review process averaged 4.5 minutes per patient (1.5 minutes to access the data in Dexcom Clarity and review it for patterns, 1 minute to log into the patient’s record and document changes, 2 minutes to send the patient a message using a secure EMR-based messaging platform). The weekly specificity, positive predictive power, and negative predictive power of TIDE are shown in Table 1.

**Table 1:** Outcomes including sensitivity identifying patients appropriate for asynchronous contact through the medical record and the reduction in the number of patients reviewed.

Week of review	February 7, 2020	February 14, 2020	February 21, 2020	February 28, 2020	March 6, 2020	March 13, 2020	March 20, 2020
Patients in study, N	56	58	59	62	64	64	64
Patients flagged by TIDE^a^, n	29	29	36	39	39	37	36
Patients reviewed, %	52	50	61	63	61	58	56
Reduction in patients reviewed^b^, %	*48*	*50*	*39*	*37*	*39*	*62*	*63*
True positive flags^c^, %	29	21	31	27	30	34	34
True negative flags^d^, %	23	17	19	15	9	16	19
False positive flags^e^, %	23	33	36	40	39	33	31
False negative flags^f^, %	2	0	0	0	0	0	2
Insufficient CGM^g^ data, %	9	10	10	6	8	9	9
Sensitivity^b^, %	*94*	*100*	*100*	*100*	*100*	*100*	*96*
Specificity, %	50	34	34	26	19	32	38
Positive predictive value, %	55	39	46	40	43	51	52
Negative predictive value, %	93	100	100	100	100	100	92

^a^TIDE: timely interventions for diabetes excellence.

^b^Primary objective (italicized).

^c^Flagged by TIDE and appropriate for asynchronous contact.

^d^Not flagged by TIDE and not appropriate for asynchronous contact.

^e^Flagged by TIDE and not appropriate for asynchronous contact.

^f^Not flagged by TIDE and appropriate for asynchronous contact.

^g^CGM: continuous glucose monitor.

### Validation on External Data Sets

There were 1424 patients with at least 1 day of CGM data that met the inclusion criteria in the 8 external data sets. There were 168,723 patient days with CGM readings across 30,076 weeks. The patient with the most included days had 1028 days over 154 weeks, whereas the patient with the fewest days had 1 day. The mean weekly ACT was 5.1 (IQR 4.0-6.33), MG was 170.7 (IQR 148.8-189.2), mean percentage of TIR 70 to 180 mg/dL was 56.6% (IQR 45.1%-68.4%), eHyp <54 mg/dL was 1.9% (IQR 0.25%-2.32%), and Hyp <70 mg/dL was 3.3% (IQR 1.50%-4.60%). Across data sets, the minimum and maximum number of patients included were 12 and 450, respectively; the number of CGM days per patient was 9.8 and 256.8, respectively; MG was 141.3 (SD 21.3) and 207.0 (SD 35.3), respectively; mean percentage TIR was 38.2% (SD 14.2%) and 74.2% (SD 13.3%), respectively; Hyp was 2.1% (SD 2.3%) and 4.6% (SD 3.8%), respectively; and eHyp was 0.5% (SD 0.9%) and 3.7% (SD 4.9%), respectively (Table 2).

**Figure 3:**
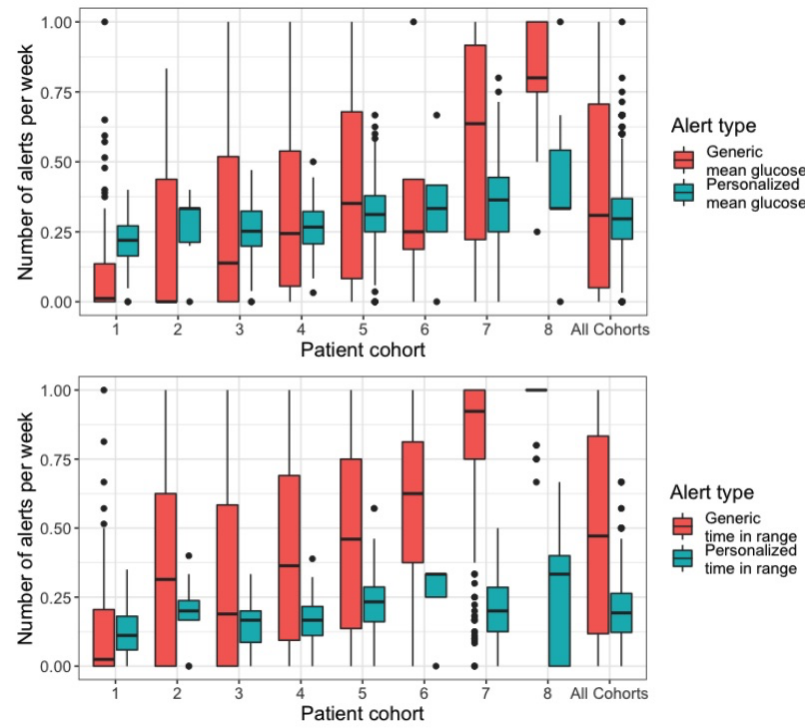
Frequency of generic and personalized flags in external cohorts.

Data sets were numbered by increasing mean MG. The analysis of personalized MG and TIR flags included 1100 patients with at least 4 weeks of data. The median frequency was significantly higher for generic TIR flags than for personalized TIR flags, 0.47 (IQR 0.12-0.83) versus 0.19 (IQR 0.12-0.26) flags per patient per week (*P*<.001), respectively, as was the SD of the frequency of flags (0.36 vs 0.11, respectively; *P*<.001; Figure 3). The median frequency of flags was significantly higher for generic MG than for personalized MG, 0.31 (IQR 0.05-0.71) versus 0.30 (IQR 0.22-0.37) flags per patient per week (*P*<.001), as was the SD of the frequency of flags 0.35 versus 0.12 (*P*<.001; Figure 3). In the two data sets with the highest mean MG, data sets 7 and 8, respectively, 49.7% (97/212) and 81% (26/32) of patients had a generic MG or TIR flag every week. Across all data sets, 15.64% (172/1100) of patients had a generic MG or TIR flag every week, and 0% (1/1100) of patients had a personalized MG or TIR flag every week (Table 3).

**Table 2:** Continuous glucose monitor (CGM) data in external data sets.

Data set	Patients, n	CGM days, n (number per patient)	CGM weeks, n (number per patient), n	Days active, mean (SD)	Glucose (mg/dL), mean (SD)	Time in range^a^ (%), mean (SD)	Percentage of time hypoglycemic^b^, mean (SD)	Percentage of time extremely hypoglycemic^c^, mean (SD)
1	85	21,236 (249.8)	3372 (39.7)	6.3	141.3 (21.3)	74.2 (13.3)	3.1 (2.4)	2 (3.1)
2	12	315 (26.2)	63 (5.2)	5	152.9 (23.3)	66.4 (13.2)	4 (3)	1.7 (2.4)
3	120	30,815 (256.8)	4546 (37.9)	6.8	158.3 (26.7)	65.9 (16)	2.1 (2.3)	0.5 (0.9)
4	225	48,805 (216.9)	7670 (34.1)	6.4	162.3 (24.1)	62.1 (14.1)	3 (2.6)	0.9 (1.4)
5	450	54,535 (121.2)	11,936 (26.5)	4.6	162.6 (28)	62.4 (16.3)	2.9 (3.1)	1 (2.3)
6	180	1682 (9.3)	378 (2.1)	4.4	176.6 (33.3)	50.4 (15.6)	3.6 (3.2)	3.4 (4.3)
7	219	10,025 (45.8)	1727 (7.9)	5.8	184.9 (44.4)	45.1 (16.6)	4.6 (3.8)	3.7 (4.9)
8	133	1310 (9.8)	384 (2.9)	3.4	207 (35.3)	38.2 (14.2)	2.9 (3.7)	1.6 (3.6)

^a^Percentage of readings that were 70 to 180 mg/dL.

^b^Percentage of readings <70 mg/dL.

^c^Percentage of readings <54 mg/dL.

**Table 3:** Frequency of generic and personalized flags based on continuous glucose monitor data in external data sets.

Data set	Patients, n	Patients with at least 4 weeks of data, n (%)	Glucose flags, n (flags per patient per week)		Time in range flags^a^, n (flags per patient per week)		Patients with mean glucose or time in range flag every week, n (%)	
			Generic^b^	Personalized^a^	Generic^c^	Personalized^d^	Generic	Personalized
1	85	78 (92)	329 (0.1)	725 (0.22)	493 (0.15)	493 (0.12)	1 (1)	0 (0)
2	12	10 (83)	13 (0.22)	14 (0.24)	19 (0.33)	19 (0.16)	1 (10)	0 (0)
3	120	120 (100)	1417 (0.31)	1128 (0.25)	1535 (0.34)	1535 (0.15)	4 (3.3)	0 (0)
4	225	225 (100)	2595 (0.34)	2002 (0.26)	3204 (0.42)	3204 (0.16)	9 (4)	0 (0)
5	450	436 (97)	4137 (0.35)	3498 (0.29)	4895 (0.41)	4895 (0.21)	31 (7.1)	0 (0)
6	180	4 (2)	6 (0.38)	4 (0.25)	9 (0.56)	9 (0.19)	1 (25)	0 (0)
7	219	195 (89)	967 (0.58)	525 (0.31)	1360 (0.81)	1360 (0.18)	97 (49.7)	0 (0)
8	133	32 (24)	116 (0.84)	47 (0.34)	131 (0.95)	131 (0.22)	26 (81.3)	1 (3.1)

^a^Personalized mean glucose (MG) flag triggered when MG>MG+10 mg/dL in baseline period.

^b^Generic mean glucose flag triggered when mean bigeneric glucose >170 mg/dL.

^c^Generic time in range flag triggered when the percentage of readings of 70-180 mg/dL was <60%.

^d^Personalized time in range (TIR) flag triggered when TIR<TIR−10% points in baseline period.

## Discussion

### Principal Findings

We designed TIDE as an open-source hardware-agnostic tool for the personalized analysis of CGM data at the clinic population scale. In a pediatric T1D clinic, TIDE identified 99% of patients appropriate for contact using an asynchronous message through the EMR while reducing the number of patients reviewed by certified diabetes educators by 43%. For each of the 8 external data sets, simulation of the use of TIDE produced fewer than 0.25 personalized TIR flags per patient per review period. TIDE and the underlying algorithms are free open-source software available on GitHub [30,31]. Upon request, we will help clinicians customize the tool to their setting and deploy it in practice.

TIDE was developed with an iterative agile approach to support asynchronous contact with patients, the form of contact found to be most effective by a systematic review of T1D telemetry systems [15]. An initial version of TIDE was produced quickly, and few significant design decisions were made before physicians and CDCESs used TIDE to provide feedback. Physicians and CDCESs identified their preferences for a rule-based approach over less-interpretable approaches such as machine learning, time-series analysis, or alternative statistical smoothing techniques. On the basis of physician and CDCES feedback, TIDE was designed to identify how a patient’s glucose management differs from validated recommendations and to produce interpretable flags to facilitate recommendations. As TIDE uses consensus guidelines, it may be more broadly applicable than a model trained on a small or nonrepresentative subset of the population would be. The use of TIDE to identify patients for asynchronous messaging fits well with the CDCES workflow. The CDCES could send a message to each patient identified by TIDE and move immediately to the next patient, rather than spending time scheduling an appointment or trying to contact the patient or family. The primary challenges of the agile approach and asynchronous messaging are that additional resources may be required to ensure that patient care is not adversely affected during the deployment of an early stage tool or because of patients ignoring messages. In this study, during the initial testing period, a physician reviewed the output of TIDE to ensure that the quality of care was not compromised, and the CDCES tracked whether patients read their messages and followed up accordingly.

The use of generic and personalized flags has complementary benefits. For patients with average glucose management, the generic flags provide a standardized approach to care based on the most recent consensus guidelines. For patients with very well or very poorly managed glucose levels, personalized flags based on patient progress may be more informative. If a person with MG 208 (the mean in one of the external data sets) consistently reduced their glucose by 5 mg/dL per review period, a generic metric may trigger a flag for numerous consecutive review periods, whereas a personalized metric would indicate improvement. During the first major revision of TIDE, a personalized metric was added to track the TIR to help CDCESs identify changes in patient management that did not cross the threshold of a personalized metric. With any flags, particularly in pediatric and young adult populations, it is important to further test the optimal timing and frequency (ie, the dose) to strike the correct balance of receiving action-oriented guidance while not further burdening the person.

Nonendocrinologists care for numerous people with T1D [32]. Clinicians who are not aware of the most recent consensus guidelines or who are not comfortable with diabetes technology, such as CGM data, may not have the resources to provide patients with appropriate care recommendations. Programs to provide telemedicine-based care or train nonspecialists are associated with better outcomes but require resources and time investment that limit participation and scope [33,34]. The use of a relatively simple tool with metrics and targets based on the consensus guidelines may be useful for nonspecialist clinicians to inform the care of such patients.

### Strengths and Limitations

A strength of this study is the evaluation across 8 external datasets of the potential applicability of TIDE. Most T1D clinics in the United States see patients 3 to 12 times per year primarily in person [3]. In this study, reviewing a patient’s data with TIDE and sending the patient a message required an average of 4.5 minutes. In a clinic in which patients use CGM and 15-minute-long in-person visits, eliminating an average of 1 in-person visit per patient per year would provide sufficient time for an average of 3 message-based contacts. Using TIDE to support such a workflow requires that TIDE flags an average of 0.25 patients per month for review, equivalent to 3 reviews per patient per year. In a simulation of the use of TIDE for populations with differing average levels of glucose management, the personalized TIR metric flagged no more than 0.22 patients per review period, even for the population with an average MG level of 207 mg/dL. As TIDE flags patients based on deteriorating glucose control, patient contacts would be targeted to address the need rather than per a fixed schedule. Each patient would be more likely to receive care when their control deteriorates. On an average, patients with worse control may receive more contacts than those with better control. Such deployments of TIDE are ongoing with two partner clinics, one in the United States and another in Australia, each caring for ≥1000 patients with T1D and using CGMs.

This study has several limitations. The workflow presented requires downloading data and toggling between the tool and Dexcom Clarity and is not integrated with the EMR. Integration of CGM data with the EMR will facilitate integration with current telehealth workflows and allow the tool to incorporate data on the timing of each patient’s previous and upcoming visits into the recommendations. The specificity of this tool was significantly lower than its sensitivity. However, specificity is less relevant than the direct measure of the primary outcome and time savings associated with the use of TIDE. Specificity may be improved by using the data on which patients did and did not require a review to tune the algorithm for which patients should be flagged. Improvements are ongoing to streamline, standardize, and scale the data review; to improve the sensitivity and specificity of the tool; and to incorporate the tool into the EMR [35]. There was a 43% reduction in the number of patients reviewed each week; however, reviewing patients weekly is not currently the standard of care and may represent increased time spent for most diabetes clinicians. The reduction in the number of patients requiring review in a different setting may change with the cadence of the review and the criteria for review. This study was conducted as a novel proof-of-concept intervention to create new knowledge and generate data on the performance of an automated tool. The metrics and thresholds were not derived from a systematic hypothesis-based approach or a survey of patients, families, and clinicians. The thresholds used to create the tool were based on consensus guidelines with the input of a group of experts in diabetes technology, and the revised version of the tool used feedback from 4 weeks of use in clinical care.

### Future Studies

Subsequent efforts to deploy TIDE in other settings may benefit from a formal quality improvement framework with an established aim, predefined targets for metrics, implementation criteria, and well-defined iteration cycles*.* Subsequent research is ongoing to explicitly incorporate measurements of the additional time necessary for clinical decision-making based on the review of the data, examine the operational requirements to expand the number of individuals monitored with the help of this tool, and identify if the use of such a tool may allow a clinic with fixed resources to provide care for more patients through a more efficient use of clinician time [35].

### Conclusions

We developed and deployed TIDE, a tool that uses metrics based on consensus guidelines, to identify 99% of patients appropriate for contact using an asynchronous message while reducing the number of patients requiring review by a physician or certified diabetes educator by 43%. Further investigation is necessary to understand the potential of automated analyses of CGM data to support broader access to personalized and timely glucose management.

## Appendix

Multimedia Appendix 1 Full list of generic metrics, sources of data for retrospective analysis, and timely interventions for diabetes excellence screenshots.

## Abbreviations

ACT: number of days continuous glucose monitor active
ADA: American Diabetes Association
CDCES: certified diabetes care and education specialist
CDS: clinical decision support
CGM: continuous glucose monitor
eHyp: extreme hypoglycemic
EMR: electronic medical record
Hyp: hypoglycemic
MG: mean glucose
T1D: type 1 diabetes
TIDE: timely interventions for diabetes excellence
TIR: time in range
