# Supplementary material for: Algorithm-Enabled, Personalized Glucose Management for Type 1 Diabetes at the Population Scale: Prospective Evaluation in Clinical Practice
Source: JMIR Diabetes. 2022 Jun 6;7(2):e27284. doi: 10.2196/27284 (PMC9210201; doi:10.2196/27284)
Supplement: Multimedia Appendix 1 [file diabetes_v7i2e27284_app1.pdf]

Population-Level Personalized Diabetes Management Facilitated by Analyses of Continuous Glucose Monitor Data and Telehealth Visits

Supplemental Table 1 – Sources of data for retrospective analysis

Metric name	Definition
Number of active CGM days (ACT)*	Number of days with minimum number of CGM readings for inclusion
First CGM day	First day with minimum number of CGM readings for inclusion
Last CGM day	Last day with minimum number of CGM readings for inclusion
**Variable timeframe metrics****	
Mean glucose (MG)	Mean of glucose readings
Percentage of time extremely hypoglycemic (eHyp)	Percent of readings < 54 mg/dL
the percentage of time hypoglycemic (Hyp)	Percent of readings < 70 mg/dL
Percentage of time in range	Percent of readings 70-180 mg/dL
Standard deviation of glucose	Standard deviation of glucose readings
Coefficient of variation	Coefficient of variation of glucose readings
Percentage of time in range, conservative	Percent of readings 70-140 mg/dL
Percentage of time with high glucose	Percent of readings > 180 mg/dL
Percentage of time with very high glucose	Percent of readings > 250 mg/dL
Mean glucose while in target range	Mean of readings 70-180 mg/dL
Mean glucose with high glucose	Mean of readings > 180 mg/dL
Standard deviation of glucose while in target range	Standard deviation of readings 70-180 mg/dL
Standard deviation of glucose with high glucose	Standard deviation of readings >180 mg/dL
Coefficient of variation while in target range	Coefficient of variation of readings 70-180 mg/dL
Coefficient of variation with high glucose	Coefficient of variation of readings >180 mg/dL

*Minimum percentage for inclusion is a modifiable parameter. It was set to 70% in this study.
**Each metric measured full day (00:00-24:00), nighttime (00:00-00:06:00), and daytime (06:00-23:59). Full day results were presented in this study.

Supplemental Table 2 – Sources of data for retrospective analysis

Cohort	Study or source	N People	Duration of continuous glucose monitor data
1	Donated by OpenAPS (https://openaps.org/)	91	Between weeks and years
2	Haymond, M.W., DuBose, S.N., Rickels, M.R., Wolpert, H., Shah, V.N., Sherr, J.L., Weinstock, R.S., Agarwal, S., Verdejo, A.S., Cummins, M.J., Newswanger, B., Beck, R.W., 2017. Efficacy and Safety of Mini-Dose Glucagon for Treatment of Nonsevere Hypoglycemia in Adults With Type 1 Diabetes. J Clin Endocrinol Metab, 102(8), pp.2994-3001.	26	Approximately 2 weeks
3	Donated by Tidepool, Palo Alto, CA	120	Between weeks and years
4	Aleppo, G., Ruedy, K.J., Riddlesworth, T.D., Kruger, D.F, Peters, A.L., Hirsch, I., Bergenstal, R.M., Toschi, E., Ahmann, A.J., Shah, V.N., Rickels, M.R., Bode, B.W., Philis-Tsimikas, A., Pop-Busui, R., Rodriguez, H., Eyth, E., Bhargava, A., Kollman, C., Beck, R.W., 2017. REPLACE-BG: A Randomized Trial Comparing Continuous Glucose Monitoring With and Without Routine Blood Glucose Monitoring in Adults With Well-Controlled Type 1 Diabetes. Diabetes Care, 40(4):538-545.	226	6 months
5	JDRF CGM Study Group. JDRF randomized clinical trial to assess the efficacy of real-time continuous glucose monitoring in the management of type 1 diabetes: research design and methods. Diabetes Technol Ther. 2008;10(4):310-321.	451	1 year
6	Weinstock, R.S., DuBose, S.N., Bergenstal, R.M., Chaytor, N.S., Peterson, C., Olson, B.A., Munshi, M.M., Perrin, A.J.S., Miller, K.M., Beck, R.W., Liljenquist, D.R., Aleppo, G., Buse, J.B., Kruger, D., Bhargava, A., Goland, R.S., Edelen, R.C., Pratley, R.E., Peters, A.L., Rodriguez, H., Ahmann, A.J., Lock, J., Garg, S.K., Rickels, M.R., Hirsch, I.B., 2015. Risk Factors Associated With Severe Hypoglycemia in Older Adults With Type 1 Diabetes Diabetes Care, Dec 2015	203	14 days
7	Bergenstal, R.M., Gal, R.L., Connor, C.G., Gubitosi-Klug, R., Kruger, D., Olson, B.A., Willi, S.M., Aleppo, G., Weinstock, R.S., Wood, J., Rickels, M., DiMeglio, L.A., Bethin, K.E., Marcovina, S., Tassopoulos, A., Lee, S., Massaro, E., Bzdick, S., Ichihara, B., Markmann, E., McGuigan, P., Woerner, S., Ecker, M., Beck, R.W., 2017. Racial Differences in the Relationship of Glucose Concentrations and Hemoglobin A1c Levels. Ann Intern Med. 167(2):95-102	232	12 weeks
8	Nwosu, B.U., Maranda, L., Cullen, K., Greenman, L., Fleshman, J., McShea, N., Barton, B.A., Lee, M.M., 2015. A Randomized, Double-Blind, Placebo-Controlled Trial of Adjunctive Metformin Therapy in Overweight/Obese Youth with Type 1 Diabetes. PLoS One, 10(9):e0137525.	139	6 months

Figure: Screen captures of TIDE

A. Initial TIDE interface

Alerts Summary | Raw Data

Show 10 entries | Search:

	Patient	Worn	Mean	TIR	TBR.54	TBR.70	Readings	5.Min.Int
1	Patient AR	93.37	183.21	50.59	0	0.88	3619	3876
2	Patient DR	95.68	126.74	81.83	0.27	4.71	3715	3883
3	Patient SJ	69.97	126.59	95.21	0	0.04	2716	3882

Showing 1 to 3 of 3 entries | Previous 1 Next

B. Revised TIDE interface

All Patients | Alerts | No Data | No Alerts

Show 10 entries | Search:

	Patient	Worn (%)	Most Recent Week TIR (%)	Previous Month TIR (%)	Change (%)	< 54 (%)	< 70 (%)
1	Patient 1	69	74	59	15	0	0
2	Patient 2	97	92	91	1	0.2	5
3	Patient 3	99	71	91	-20	0	1
4	Patient 4	91	77	68	9	0.6	8
5	Patient 5	93	43	47	-4	0	1
6	Patient 6	87	87	79	8	0.91	9
7	Patient 7	96	60	56	4	0.88	5
8	Patient 8	88	50	41	9	0.34	2
9	Patient 9	98	74	80	-6	1.02	4
10	Patient 10	91	94	80	14	0.22	1

Showing 1 to 10 of 23 entries | Previous 1 2 3 Next
